# Porous Copolymer Resins: Tuning Pore Structure and Surface Area with Non Reactive Porogens

**DOI:** 10.3390/nano2020163

**Published:** 2012-06-06

**Authors:** Mohamed H. Mohamed, Lee D. Wilson

**Affiliations:** 1 Aquatic Ecosystems Protection Research Division, Water Science and Technology Directorate, 11 Innovation Boulevard, Saskatoon, Saskatchewan, S7N 3H5, Canada; Email: mohamed.mohamed@usask.ca; 2 Department of Chemistry, University of Saskatchewan, 110 Science Place—Room 165, Thorvaldson Building, Saskatoon, Saskatchewan, S7N 5C9, Canada

**Keywords:** porous copolymer resins, porogen, suspension polymerization, porosity, surface area

## Abstract

In this review, the preparation of porous copolymer resin (PCR) materials via suspension polymerization with variable properties are described by tuning the polymerization reaction, using solvents which act as porogens, to yield microporous, mesoporous, and macroporous materials. The porogenic properties of solvents are related to traditional solubility parameters which yield significant changes in the surface area, porosity, pore volume, and morphology of the polymeric materials. The mutual solubility characteristics of the solvents, monomer units, and the polymeric resins contribute to the formation of porous materials with tunable pore structures and surface areas. The importance of the initiator solubility, surface effects, the temporal variation of solvent composition during polymerization, and temperature effects contribute to the variable physicochemical properties of the PCR materials. An improved understanding of the factors governing the mechanism of formation for PCR materials will contribute to the development and design of versatile materials with tunable properties for a wide range of technical applications.

## 1. Introduction

### 1.1. Aim

Since their emergence, porous copolymer resins (PCR) have been used in various applications including sorption [1,2,3], solid supports in catalysis [4,5], chemical separations [6], ion-exchange [7,8], chromatography [7,8] and other applications. The usage of PCR materials is related to the unique physicochemical properties and the porous structural features of the polymer resins. Some examples of pore design include foaming (use of gaseous porogens), phase separation (use of solvent porogens), emulsions (high internal phase emulsions), and template synthesis (molecular imprinting) [9].

This review will focus on the phase separation technique where vinyl and acrylic PCR-based materials are synthesized from acrylates, styrene, divinylbenzene, and vinyl pyridine via suspension polymerization, in the presence of single and/or multi-component solvent porogens [2,4,6,7,10,11,12,13]. Suspension polymerization [14] is a technique where monomer(s), relatively insoluble in water is (are) dispersed as liquid droplets with a steric stabilizer that acts to hinder the coalescence of monomer droplets and the adhesion of partially polymerized particles during polymerization, using vigorous stirring to produce polymer particles as a dispersed phase. A porogen is either a low molecular weight compound [1,2,4,5,7,11,12,13,14] or a polymer [15,16,17] that is miscible with the monomers. The porogen is inert and can be readily removed, resulting in voids after the reaction with the formation of porous polymeric materials. The term “pore-forming agents” [9,10,14,15,16,17,18,19,20,21,22,23] is sometimes used instead of “porogens”.

### 1.2. Pore Formation Mechanism

**Figure 1:**
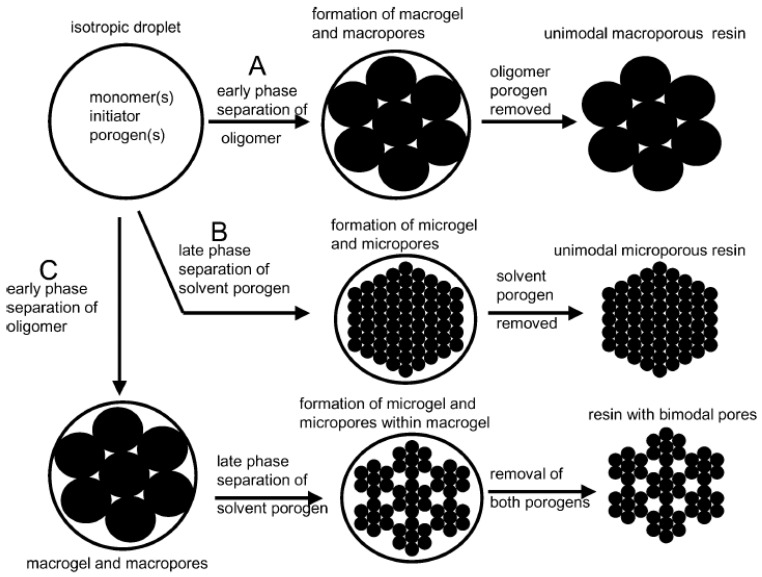
Schematic representation of pore formation in poly(DVB) resins: (**A**) using a “bad” solvent porogen; (**B**) using a thermodynamically “good” solvent porogen; and (**C**) using a mixture of “good” and “bad” solvent porogen as co-porogens. (Reprinted (adapted) with permission from [18]. Copyright 2004 American Chemical Society). NB: The pores are of different sizes and are not drawn according to scale, as shown below.

The general mechanism of how porogens control the resin surface area, pore-size, pore-size distribution (PSD), pore-volume, and morphology is illustrated in Figure 1 [18]. The period of phase separation, shown in Figure 1, is the main rate determining step. As a general rule, phase separation will occur at a much later stage if the porogen is a “good” solvent or a solvating diluent [24], *i.e*., miscible with both the monomer and its resulting polymer. On the other hand, the “bad” solvent or non-solvating diluent has poor miscibility at the polymer level. If a non-solvating diluent is used (A in Figure 1), microgels [2,18] will form, fuse together, and aggregate into larger clusters. This results in an increased volume of the voids (pores) forming larger pores (*i.e*., macropores) and reduced polymer surface area. Unlike non-solvating diluents, a solvating diluent (B in Figure 1) delays phase separation; hence, ensuring that microgels retain their individuality even though they undergo fusion. This leads to a reduced distance between voids or clusters; hence, the pore-sizes are reduced (mesopores and micropores) and the polymer surface area increases. The mixture of A and B in Figure 1, offers two modes of phase separation, *i.e*., early and late. Thus, bimodal pores are formed as follows: micropores/mesopores or mesopores/macropores or micropores/macropores. It is noteworthy that many authors use the term “*good thermodynamic solvent*” instead of “*solvating diluent*”. In this review, the term *solvating diluent* is preferred because the thermodynamic parameters of these solvents are not adequately characterized in the context of PCR materials; therefore, the term “*good thermodynamic solvent*” was not used in view of the existing knowledge gaps (*vide infra*), and to avoid misleading the reader that a thermodynamic analysis of solvation was reviewed herein. 

### 1.3. Review Motivation

The allure towards this technique is demonstrated by the variation in the surface area (SA), pore-size, pore-size distribution (PSD), pore-volume, and morphology. The SA is found to vary from ~10^1^–10^3^ m^2^/g, PSD ranges from microporous, mesoporous and macroporous with unimodal and bimodal distributions. Pore-volumes typically range from ~0–3 cm^3^/g [1,2,4,5,7,11,12,13,14,15,16,21,22] where the higher SA values are due to solvating diluents. The average pore diameter and PSD may also vary, *i.e*., increase, decrease, or both. Moreover, the morphology coarsens in a less subtle fashion when a non-solvating diluent is used, and does not coarsen or becomes more refined in the case of a solvating diluent [2,25,26].

Some common types of porogens investigated include organic solvents of variable molecular weight. Examples of low molecular weight solvent porogens include toluene, hexane, cyclohexanone, 2-ethylhexanol, *p*-xylene, *n*-heptane [2,4,15,16] and an example of a higher molecular weight solvent is poly(ethylene glycol) [15,23]. On the other hand, inorganic porogens include sodium chloride [24,25,26], silica [25], sodium hydrogen carbonate [27] and supercritical carbon dioxide (SCD) [25,26,27]. SCD is a suitable porogen substitute for other types of solvents that are otherwise challenging to remove from the polymer matrix at the purification stage and/or polymers which are sensitive to very small composition changes of the porogenic solvent mixtures. SCD offers fine control over average pore-sizes and PSD using temperature and pressure conditions to alter the density of SCD. The popular use of organic porogens is traced back to the 1950s [9]; whereas, the first example of SCD as a porogen for modifying PCR materials was more recently reported by Holmes and Cooper [28,29,30].

PCR tuning with porogens has not been extensively studied in a systematic manner and is the subject of this review. This paper describes the surface area, pore-size, PSD, pore-volume and changes in the morphological properties when polymers are tuned with porogenic “inert” solvents at various conditions. In addition, knowledge gaps related to this technique will be addressed and some recommendations for future research are suggested herein.

### 1.4. Criteria for Porogen Selection

The criteria for choosing porogenic solvents is often based on their respective molecular size [24], alky chain length [31], mixtures of lipophilic and hydrophilic porogens [32], and solubility parameter (SP) [33,34,35,36,37]. The SP was identified as a quantitative criterion for predicting the pore structure properties of polymers; however, the SP was not adequately addressed in other studies [12,37]. There are three general types of SP values, *i.e*., a one-component SP is also referred to as the Hildebrand solubility parameter, a two-component solubility (physical-chemical) parameter, and the Hansen’s three-component solubility parameter [31,32,33,34,35,36].

The three approaches employing SP criteria have been applied in various studies. In view of its simplicity, the Hildebrand one-dimensional SP is used for solutions without molecular polarity or specific intermolecular interactions. The two-dimensional SP is preferred over the Hansen’s three-dimensional SP for solvents with lower molar volumes since they yield comparable results. Hansen’s three-dimensional SP is favored [33,36] for solvents with greater molar volumes, such as phthalates, and provides reasonably accurate results.

The tuning of PCR materials with the aforementioned porogens is determined by various factors [2,4,6,7,10,15,16,17,18,19,20,21,38], as follows: (*i*) the feed ratio of the porogen to monomer mixture (or porogen volume) yields optimal conditions when the ratio is maximized; (*ii*) porogens with variable SP; (*iii*) mixing multiple porogens, *i.e*., co-porogens; (*iv*) variable temperatures where higher temperatures produce smaller pores; (*v*) variable stirring speeds influence microemulsion formation and the particle size; (*vi*) crosslinking density affects the PSD; (*vii*) polymer composition where the relative monomer type and feed ratio affects the pore-size and PSD; and (*viii*) the nature of the polymerization technique. Baydal *et al. *[39] investigated the influence of different parameters and found that the type of porogen and the relative volume have a large influence on the copolymer resin properties. Based on their findings, the goal of this review focuses on tunable porogens of variable types, both as pure solvents and their mixtures (co-porogens). In addition, the influence of porogen volume is examined while the other factors are treated as secondary in their importance.

## 2. Synthesis and Characterization

### 2.1. Synthesis

As mentioned above, this review covers the topic of suspension polymerization as the main synthetic approach for preparing PCR materials. A schematic representation of this polymerization technique was described previously (*cf*. Figure 8 in [40]). In general, there is an organic phase (also referred to as the discontinuous phase) comprised of predetermined amounts of monomer(s), initiator, and solvent(s) which are added into an aqueous phase, and is also referred to as the continuous phase. The mixture is comprised of a suspension stabilizer in water in a reactor vessel at ~60–70 °C with mechanical stirring at a suitable speed for a desired particle size. The reaction is maintained ~80 °C for 3–24 hours and the resulting spherical particles are washed with water, ethanol, or methanol, and extracted with a suitable solvent such as acetone for 24–48 hours [1,2,4,5,7,11,12,13,14,15,16,17,18,19,20,21,22,23,24,25,26,27,28,29,30,31,32,33,39,40]. A commonly used polymerization initiator agent is α-α’-azo-bis-isobutyronitrile (AIBN).

### 2.2. Characterization

#### 2.2.1. Porosimetry

According to the International Union of Pure and Applied Chemistry (IUPAC), pores are classified into three categories according to their pore size: micropores (less than 2 nm), mesopores (2 to 50 nm), and macropores (larger than 50 nm). In the solid state, nitrogen porosimetry [2,4,7,10,15,16,17,38,41] is used to characterize micro- and mesopores in terms of specific surface area. The determination of the mesopore and macropore characteristics is evaluated on the basis of pore-volume and pore-volume distribution using mercury intrusion porosimetry [4,15,16,26,39,42].

#### 2.2.2. Solvent Uptake

The quantification of solvent uptake such as heptane, methanol, and toluene by the polymeric resins provide an indirect measurement of the accessible pore-volume and confirms the estimates obtained from porosimetry [4,34,41] methods. The measurement of pore-volume in the solid state using porosimetry utilizes various backfill gases in which fixed inaccessible pores are evaluated using BET analysis. In contrast, the uptake of solvent enables access to restricted pores through morphological changes (*i.e*. swelling) by filling the fixed pores, expanding pores through uncoiling of framework units, and swelling of highly crosslinked domains.

#### 2.2.3. Microscopy

Many studies have examined the use of scanning electron microscopy (SEM), transmission electron microscopy (TEM), and atomic force microscopy (AFM) to identify the morphology and texture of the polymers [2,6,7,17,26,30,43,44].

## 3. Solubility Parameter

Research concerning PCR materials utilizes the SP as a relative measure of “good” or “bad” solvents, according to the solvating ability of a diluent. There are three types of SP values that are commonly used, *i.e*., the one-component SP (the Hildebrand solubility parameter), a two-component solubility (physical-chemical) parameter, and Hansen’s three-component SP value [30,31,32,33,34,35].

The Hildebrand SP (*δ*) of the porogen and the polymer [36], respectively, are defined as shown in Equation (1) below:


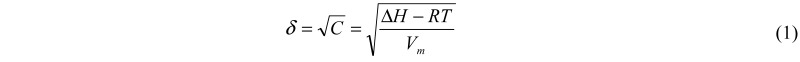


where *C* is the cohesive energy density, Δ*H* is the heat of vaporization, *R* is the gas constant, *T* is the temperature, and *V_m_* is the molar volume of the solvent. The SI unit for *δ* is MPa^1/2^ while the conventional unit is (J/cm^3^)^1/2^. The closer the match between the porogen and the polymer implies a “good” solvent since solvents with similar SP values are generally miscible. In other words, if (|Δ*δ*|) is near zero (where |Δ*δ*| = |*δ*_resin_- *δ*_solvent_|), then miscibility is favored. In cases where |Δ*δ*| > 3 MPa^1/2^, the solvent is considered “bad” for values between 1-3 MPa^1/2^, the solvent possesses intermediate solubility and is considered “good” and “bad”. However, this criterion has some exceptions when the resin and diluent are both polar or have specific polar-directional group interactions. Therefore, an extension to Hildebrand’s theory was proposed by Hansen [32] where the SP was divided into three contributions, *i.e*., dispersion forces (*δ_d_*), dipolar interactions (*δ_p_*) and H-bond capacity (*δ_h_*), as shown in Equation (2).


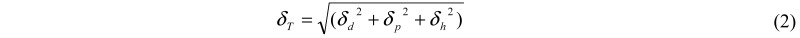


**Figure 2:**
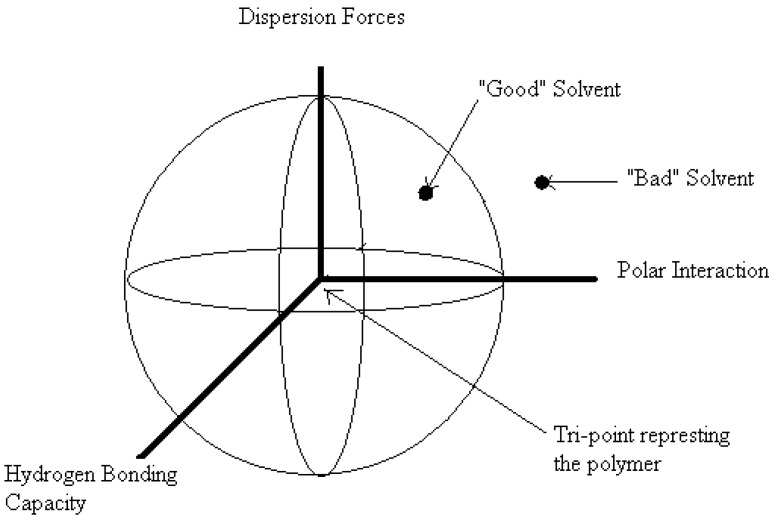
A three-dimensional plot of the three solubility parameter (SP) contributions *i.e.*, dispersion forces (*δ_d_*), dipolar interactions (*δ_p_*), and H-bond capacity (*δ_h_*), to the total SP (*δ_T_*). The tri-point represents a hypothetical polymer. Interaction radius is the shortest distance between the tri-point and the solvent.

The above three-dimensional SP (*δ_T_*) is assumed to be a vector sum where the three components are treated as solubility coordinates. Hansen reported resin solubility values using a three-dimensional model and concluded that doubling of the dispersion forces (*δ_d_*), a spherical volume of solubility would be formed for each resin. The sphere is described in Figure 2 where the SP value of a given compound can be visualized as a fixed point and referred to as a tri-point in a three-dimensional space. The coordinates of the tri-point are located by means of three SP values (*δ_d_*, *δ_p_* and *δ_h_*). When the resin is mixed with a single solvent or multiple solvents with SP values lying within the sphere, the compatibility is considered “good” while those solvents lying outside the sphere are considered “bad” (*cf*. Figure 3 in [45]).

**Figure 3:**
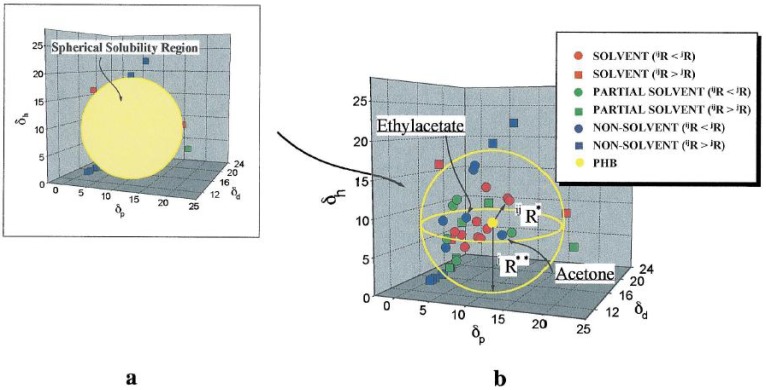
Spherical region characterizing poly(3-hydroxybutyrate) (PHB) solubility in various solvents: (**a**) the outline of the region; and (**b**) the inside of the region. The shapes indicating the solvents depend on whether the point is located inside or outside the solubility region: square and circle represent a position outside the region (^j^R < ^ij^R) and one inside the region (^j^R > ^ij^R), respectively. ^ij^R, distance of solvents at position (δ_d_, δ_p,_ δ_h_ ) from center of solubility sphere for PHB, calculated from Equation (4). (Reprinted from [45], Copyright 1999, with permission from Elsevier).

To aid the SP criterion of Hansen’s methodology for the selection of “good” versus “bad” solvents, the use of relative energy difference (*RED*) was proposed, as shown in Equation (3). In this approach, values of *RED* < 1 imply that the solvent is “good” or the solvent is “bad” when *RED* > 1.


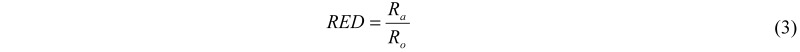


where *R_a_* is the distance between centre of the polymer solubility sphere for the solvent-solvent system and *R_o_* is the radius of the sphere; where R_a_ is calculated according to Equation (4).


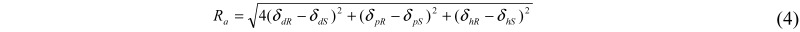


where *δ_xR_* and *δ_xS_* are the Hansen solubility parameters for the resin and solvent, respectively. The methodology used for the above criterion is not followed unanimously by all authors because an alternate method directly compares the values of R_a_ to R_o_ without calculating *RED* [34]. In some cases, Hansen’s three-dimensional approach is simplified with a two-dimensional coordinate system. This method argues that the SP values (*δ_d_* and *δ_p_*) are similar and may be incorporated as a unified parameter referred to as a volume-dependent SP (*δ_v_*) value, as shown in Equation (5) [37]. Hence, *δ_v_* – *δ_h_* graphs are used to represent polymer-solvent interactions because this methodology is preferred for practical applications, and overcomes the use of three-dimension graphical representations.


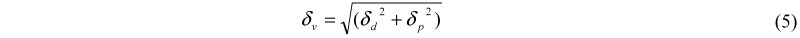


## 4. Pore Formation Mechanism

In order to have an improved understanding of the research describing the influence of solvents as porogens, the mechanism will be described in further detail. The general aspects of the mechanism are described in Figure 1. As the suspension polymerization process proceeds, the copolymer precipitates within emulsion droplets and forms spherical shapes, referred to as insoluble nuclei. The droplets form due to the relative difference in SP between the copolymer and the solvent (|Δ*δ*|). The nuclei transform into microspheres or microgel phases, and the microspheres agglomerate with each other to form a primary network. Upon further polymerization, the primary network becomes a crosslinked porous network. The phase containing solvent strongly contracts in volume due to the loss of solvating co-monomers; thereafter, network formation and phase separation occurs. As the porogen is removed, the void spaces that remain are referred to as the pores in the polymer network. The pore sizes depend on the solvating ability of the porogen. If the porogen is a solvating diluent, *i.e*., where |Δ*δ*| is close to zero, the polymer chains remain dissolved in the mixture for a longer time prior to phase separation. As the microsphere particles undergo aggregation, they are likely to retain their microparticle nature; thereafter, resulting in smaller pores. As the value for |Δ*δ*| becomes larger for a given porogen, the microsphere fails to retain its individuality and undergoes aggregation into larger clusters. The widening of the voids between subunits results in pore formation in the macropore range (*cf*. Figure 15 in [40]).

## 5. Influence of the Solvent (Porogen)

### 5.1. Pure Solvents

Research results on the influence of pure solvents as porogens for different resins are summarized in Table 1 [10,15,16,46]. Table 1 illustrates how the surface area varies in accordance with the use of different porogens. Additionally, the results show how a similar class of resins may be tuned to form micro-, meso- and macropores [7,12,44,45]. As mentioned above, solvents that act as solvating diluents may generate micropores; thereby, increasing the polymer surface area.

For example, the results from reference [16] in Table 1 show that toluene is a solvating diluent and cyclohexanone is a non-solvating diluent. 2-ethylhexanol is an intermediate case between solvating and non-solvating diluent. The results were explained by the trend for the SP values; toluene (18.2 MPa^1/2^), 2-ethylhexanoic acid (19.4 MPa^1/2^), and cyclohexanone (20.3 MPa^1/2^); whereas, the resin is ~17–18 MPa^1/2^. According to the SP values, toluene has a closer match to the resin, and this is based on the one-component SP using Hildebrand solubility parameters. However, this criterion has a fault which is illustrated in Table 1 (*cf*. [46]). The authors argue that 1-chlorodecane is a non-solvating diluent for the resin and it yields a greater surface area over the solvating diluents, demonstrating its use as a novel porogen. However, if one looks at the SP value of the solvents investigated, one observes a variation in the SP values; heptane (15.3), cyclohexane (16.8), 1-chlorodecane (17.0), toluene (18.2), and dibutyl phthalate (23.3 MPa^1/2^). The use of a one-component SP criterion to explain the results, *i.e*., if |Δ*δ*| ~ 0, the solvent is the solvating diluent and 1-chlorodecane should not have the greatest surface area. Cyclohexanol should have a surface area <25 m^2^/g when compared to the results from [2]. In contrast, Rabelo and Coutinho [34] have illustrated that a pure solvent can mislead the interpretation of results; hence, Hansen’s three-component SP value may be used. An example of this approach will be described further in Section 7.

**Table 1:** Surface area (from nitrogen porosimetry), pore-volume, pore-size and average pore diameter of porous copolymer resin (PCR) synthesized in presence of different types of solvent porogens. The synthesis properties are the same as indicated in Section 2.1.

Polymer type	Porogen	Surface area (m^2^/g)	Pore volume (cm^3^/g)	Porosity(%)	Average pore diameter (nm)	Reference
Poly(styrene- *co*-divinylbenzene) – 1:3	Cyclohexanone	25	0.22	NM ^*^	ND ^+^	[16]
	2-ethylhexanol	242	1.13		20–45	
	Toluene	599	0.44		<4	
Glycidyl methacrylate- *co*-trimethylolpropane trimethacrylate – 1:1	Octan-2-one	174	1.31	NM ^*^	10–100	[15]
	n-butyl acetate	170	1.27			
	*p*-xylene	139	1.50			
	Toluene	145	1.02			
	Ethyl acetate	110	0.66			
	Benzonitrile	<1	0.07			
	Cyclohexanone	0.2	0.16			
	Dodecan-1-ol	ND ^+^	ND ^+^			
Poly(hydroxyethyl methacrylate- *co*-ethylene glycol dimethacrylate) - 1:20	Cyclohexanol	250	0.396	NM ^*^	6.3	[10]
	Dodecanol	28	0.06		8.6	
Poly(divinylbenzene); mixtures of isomers	Heptane	482	NM ^*^	45.3 ^a^	6.9 ^a^	[46]
				69.0 ^b^	86.8 ^b^	
	Cyclohexane	640	NM ^*^	37.9 ^a^	3.8 ^a^	
				47.7 ^b^	72.4 ^b^	
	1-chlorodecane	720	NM ^*^	56.3 ^a^	7.2 ^a^	
				66.7 ^b^	53.4 ^b^	
	Toluene	701	NM ^*^	35.9 ^a^	3.2 ^a^	
				50.5 ^b^	68.1 ^b^	
	Dibutyl phthalate	661	NM ^*^	37.1 ^a^	3.6 ^a^	
				61.8 ^b^	60.2 ^b^	
	Cyclohexanol	493	NM ^*^	29.1 ^a^	3.3 ^a^	
				47.6 ^b^	77.8 ^b^	

NM ^*^ = Not Measured, ND ^+^ = Not Detected, a = Nitrogen Porosimetry, b = Mercury Porosimetry.

### 5.2. Solvent Mixtures

The preparation of PCR materials using polar co-monomers that do not dissolve in the solvent are the most solvating diluents. The use of non-solvating diluent affects the nature of the products. In this case, the most solvating diluent is mixed with the non-solvating or an intermediary solvating diluent. The prediction of the SP value is shown in Equation (6) [46].


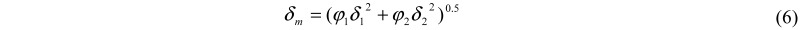


where *φ*_1_ and *φ*_2 _are the volume fractions of the two porogens, and δ_1_and δ_2 _are the respective SP values. Tuning is done by mixing the two solvents at different volume fractions until the desired properties are achieved. Table 2 shows some of the examples studied for such mixtures. Reference [16] shows that a non-solvating medium such as dimethylformamide (DMF; *δ* = 24.8 MPa^1/2^) was used as a diluent and mixed with 2-ethylhexanoic acid to yield a higher surface area material with a unimodal size distribution of 20 nm. The product obtained from toluene mixtures has a decreased surface area relative to pure toluene (*cf*. Table 1). To this point, it is assumed that a one-component SP value may explain differences in the product characteristics; whereas, the SP values of mixtures are calculated using Equation (6). The respective values are for 2-ethylhexanol:DMF (21.2 MPa^1/2^) and toluene/DMF (20.4 MPa^1/2^). The difference in SP values (|Δ*δ*| > 2) obtained using the high end of the SP scale for the resin indicates polymer formation is not favoured with high surface area and reduced pore size. The importance of using Hansens’s three-component SP is further illustrated by the results obtained for a toluene/DMF mixture. The result of adding two poor H-bonding diluents together describes why DMF contributes to an attenuation of the favourable SP of toluene, as evidenced by a decrease in the surface area of the resin. In the case of the 2-ethyhexanol mixtures, the authors conclude that the SP value increases, as compared to pure toluene and 2-ethyhexanol. The results indicate an increase in the SP value of DMF. This observation highlights one of the knowledge gaps outlined in Section 7 and Section 8. There is a pronounced effect on the matching of the SP value of the co-monomers rather than the resulting polymeric materials.

**Table 2:** Surface area (from nitrogen porosimetry), pore-volume, pore-size and average pore diameters of PCR materials synthesized in presence of various solvent mixtures. The synthesis conditions were outlined in Section 2.1.

Polymer type	Porogen A	Surface area (m^2^/g)	Pore volume (cm^3^/g)	Average pore diameter (nm)	Reference
Poly(styrene- *co*-divinylbenzene) – 1:3	2-ethylhexanol: DMF (70%:30%)	375	NM ^*^	20.0	[16]
	Toluene: DMF (70%:30%)	555		NM ^*^	
Glycidyl methacrylate- *co*-trimethylolpropane trimethacrylate – 1:1	Cyclohexanone : Dodecan-1-ol (9:1)	140	1.13	10–100	[15]
2-vinylpyridine- *co*-styrene-*co*-divinylbenzene – 1:20	Heptane : Toluene (100%:0%)	ND ^+^	ND ^+^	ND ^+^	[10]
	Heptane : Toluene (70%:30%)	131	0.5845	21.9	

NM ^*^ = Not Measured, ND ^+^ = Not Detected.

### 5.3. Solvent Volume

**Figure 4:**
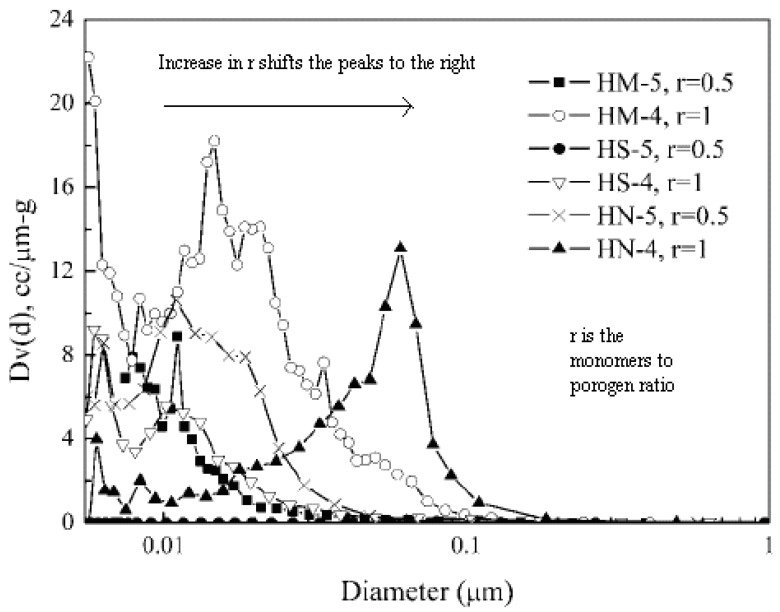
Pore size distribution of porous HM-poly(2-hydroxyethyl methacrylate-*co*-methyl methacrylate), HS - poly(2-hydroxyethyl methacrylate-*co*-styrene), and HN-poly(2-hydroxyethyl methacrylate-*co*-*N*-vinyl-2-pyrrolidone) particles. r is the ratio of monomer to porogen. PSD shifts to the right side when r increases. (Reprinted (adapted) with permission from [46]. Copyright 2008 American Chemical Society).

The volume of the porogenic solvent plays a major role in the structure and morphology of the resins. Previous research indicates that the copolymer resin prepared in the presence of larger amounts of porogen have a looser structure than those obtained with reduced levels of porogen [39,43,44,45]. In terms of pore formation, Figure 4 [42] illustrates three types of resins produced with higher porogen levels which result in a shift of the PSD toward larger pores (to the right) with greater pore abundance (higher peaks). The resin HS (poly(2-hydroxyethyl methacrylate-*co*-styrene)) has particles with smaller pores; whereas, HN (poly(2-hydroxyethyl methacrylate-*co*-*N*-vinyl-2-pyrrolidone)) particles possess larger pores. Therefore, pore size may be controlled by adjusting the relative porogen ratio. The authors conclude from their results that a lower porogen volume ratio generates fewer pores with a reduced pore size; whereas, a higher volume ratio of porogen contributes to greater numbers of pores with greater pore size. These results are further supported by Liu *et al*. [46] where greater concentration of 1-chlorodecane reveals that the precipitated phase exhibits limited swelling and the polymer aggregates behave as an immiscible “solid” polymer, similar to bulk microspheres prepared in the absence of porogen. The resulting final pore volume between aggregates should be close to the volume of the non-solvating porogen. In contrast, low levels of 1-chlorodecane yield a precipitated phase that undergoes swelling with a final pore volume less than that of the non-solvating porogen. The relative tuning of solvent porogen composition represents a good technique for tuning PSD.

### 5.4. Solvent Type

There are examples of high molecular weight organic porogens and inorganic systems; however, this review is focused on low molecular weight organic solvents in view of the continued research interest in this area. Examples of inorganic porogens of interest include sodium chloride, sodium hydrogen carbonate, silica and supercritical carbon dioxide (SCD). SCD is treated as an inorganic porogen similar to the definition of other carbon halides, sulfides, and oxides as inorganic compounds. SCD is an interesting porogen with versatile tuning capability due to its unique thermodynamic characteristics. Thus, this review will focus on SCD relative to the other types of inorganic porogens.

#### 5.4.1. Organics

##### 5.4.1.1. Low Molecular Weight

Low molecular weight porogenic solvents are mostly used due to the formation of small pores which result in products with high surface area, in addition to their relative ease of removal and low viscosity of their mixtures with monomers [2,4,6,7,9,10,11,12,13,14,15]. The opposite is true for high molecular weight porogens [18,26] and the influence of such porogens was highlighted in Sections 5.1 to Section 5.3.

##### 5.4.1.2. High Molecular Weight

**Figure 5:**
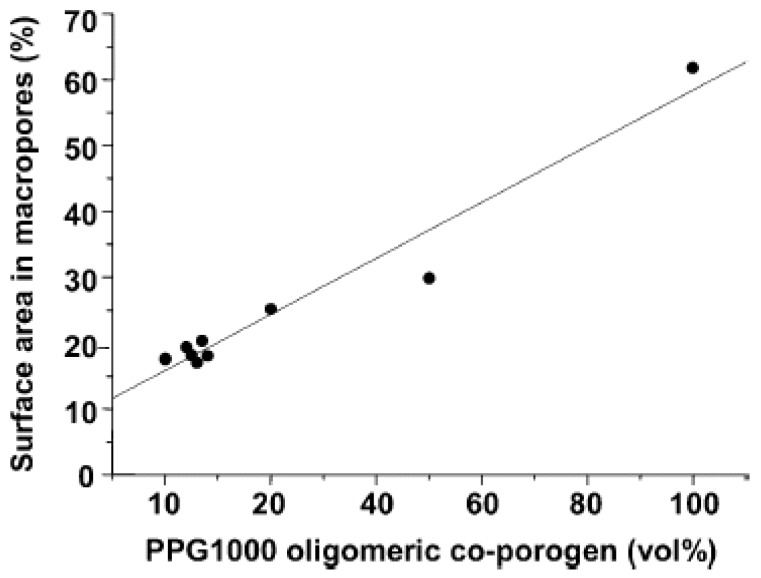
Plot of the surface area of macropores (%) *vs.* PPG-1000 co-porogen content (vol %) in the dry state for poly(DVB) resins. (Reprinted (adapted) with permission from [18]. Copyright 2004 American Chemical Society).

**Figure 6:**
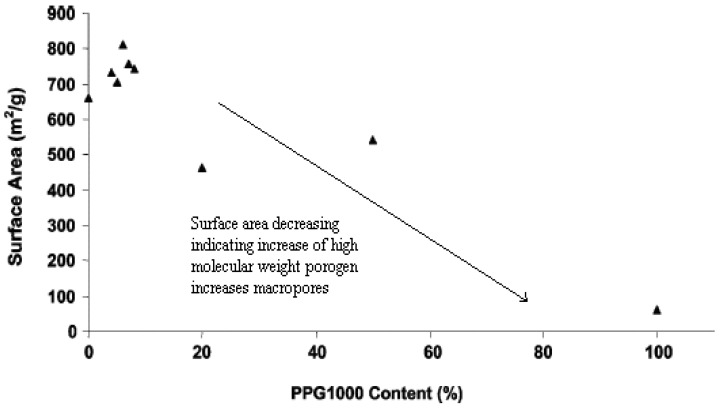
Plot of surface area (N_2_ BET) *vs.* PPG-1000 co-porogen content (vol %) for dry state poly(DVB) resins showing a maximum ~6 vol% PPG-1000. (Reprinted (adapted) with permission from [18]. Copyright 2004 American Chemical Society).

The viscosity of mixtures of monomers increases sharply with an increase in the porogen molecular weight. This affects the dispersion of droplets during polymerization and even though larger pore sizes were achieved, the pore volumes were relatively low [18]. Therefore, these porogens are commonly used with a combination of low molecular weight porogens while reducing the polymer porogen to the oligomer size (~5 kDa) regime. An example [18] using poly (propylene glycol) (PPG) with a molecular weight of ~10^3^ Daltons (Da) is mixed with toluene in a divinylbenzene resin. The surface area of the macropores (from mercury porosimetry) and the micropore/mesopore surface area (from nitrogen porosimetry) relationship are shown in Figure 5 and Figure 6 illustrating results for mixtures of PPG and toluene at different ratios [18]. The trend observed in Figure 4 supports the idea that PPG undergoes phase separation from the resin at early stages resulting in the formation of larger pores and an increase in the surface area of the macropore domains. Surprisingly, the surface area of the micropores/mesopores increase with the addition of small amounts of PPG, as shown in Figure 5. The authors conclude that PPG is unlikely to increase the proportion of small pores and the surface area. A possible explanation is that small pores were already present in the polymer matrix but were inaccessible. The addition of PPG results in larger pores with improved connectivity of the small pores. The use of PPG ~10^3^ Da yields a macroporous structure at 100% dilution; whereas, the mixture with toluene yields a mesoporous structure. The use of porogens such as PPG ~4 kDa and poly(dimethylsiloxane) >4 kDa yield polymeric products with an increase in pore size.

#### 5.4.2. Inorganics

As stated earlier, various inorganic porogens have been studied such as sodium chloride, silica and sodium hydrogen carbonate but with limited application [22,24,46]. However, SCD [25,26,27,46] is a particularly interesting inorganic porogen. SCD serves as an alternate type of porogen as compared with conventional solvent porogens that are often challenging to remove from the polymer matrix, polymers that are sensitive to very small changes in the composition of the porogen mixture, or polymeric materials that are highly compressible. Therefore, SCD offers fine control over average pore-sizes and PSD by altering the density of SCD through pressure-temperature conditions.

**Figure 7:**
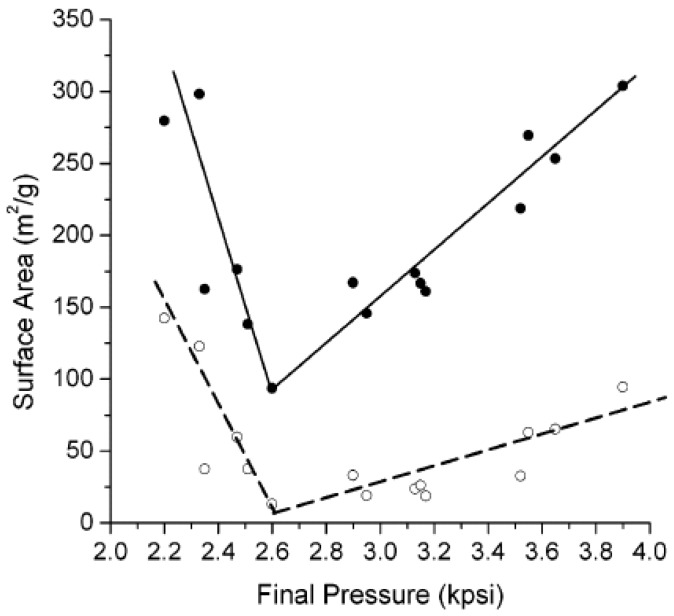
Variation in the total BET surface area (●) and micropore surface area (о) of trimethylolpropane trimethacrylate as a function of the CO_2_ final pressure. (Reprinted (adapted) with permission from [30]. Copyright 2004 American Chemical Society).

**Figure 8:**
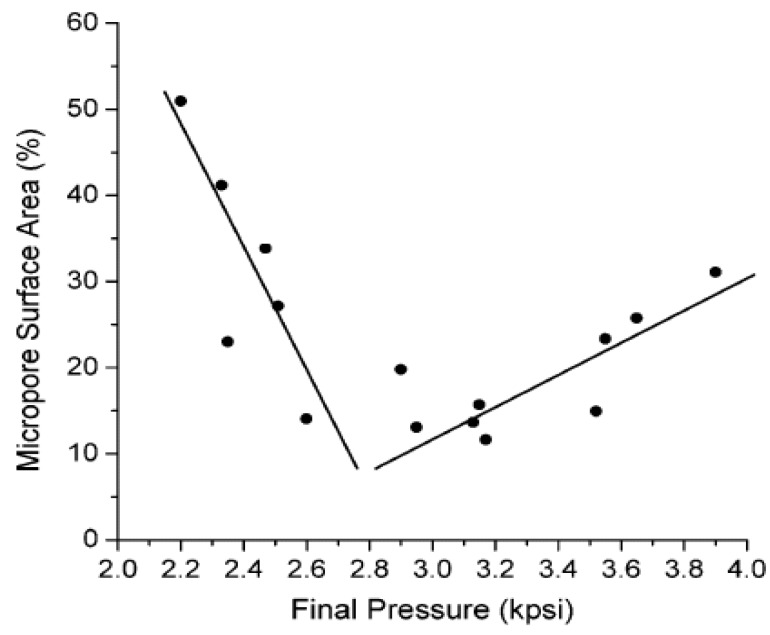
Variation of the percentage of micropore surface area (*i.e*., micropore area/total surface area × 100) of trimethylolpropane trimethacrylate as a function of the final pressure of CO_2_. (Reprinted (adapted) with permission from [30]. Copyright 2004 American Chemical Society).

Cooper and coworkers [28,29,30] are the pioneers of this methodology, as evidenced by their studies on the effect of SCD; where suspension polymerization is carried out in a pressurized reactor containing liquid carbon dioxide. All the other variables are kept constant and only the pressure is varied. The maximum pressure is restricted to the type of reaction vessel in use. The final pressure is variable because (*i*) the thermodynamics of polymerization (*i.e*., exothermic *vs*. endothermic) affect the pressure conditions, (*ii*) thermal equilibrium may not be have been met at the beginning of the reaction, (*iii*) some degree of polymerization may have occurred and (*iv*) the solutions are not ideal which was confirmed by the variation of change in molar volume (mixing of co-monomers and liquid carbon dioxide) as a function of initial pressure conditions. The porogen was removed through depressurization of the reaction mixture because of its unique P-T behaviour. *In situ* supercritical fluid extraction can be used to remove residual monomers where the relative surface area and micropore structure varies with changes in the final pressure, as shown in Figure 7 and Figure 8 [30].

Figure 7 shows that the total BET and micropore surface area decreases at pressure conditions of 2200–2600 psi and increases between 2600–4000 psi. The same trend is observed in Figure 8 for the percentage of micropore surface area relative to the total surface area. The results indicate that phase separation of SCD from the resin occurs at early stages for conditions between 2200–2600 psi. The opposite case is true between 2600–4000 psi and is supported by the results for conventional solvent porogens, as outlined in Section 5.1 to Section 5.3. According to the authors, the solvation strength of carbon dioxide increases due to an increase in pressure ~ 2600–4000 psi, due to changes in its density.

Moreover, the overall solvent properties of SCD change with an increase in pressure similar to the change in conventional solvent properties, as described in Section 5.1, and parallels the change in the properties of the resins. The exclusive relationship of an increasing SP of SCD with an increase in pressure is limited in its interpretive value, as indicated by Hamedi and Danner [47]. The relationship does not account for the changes in surface area with an increase in pressure conditions. The surface area will increase or decrease in one direction with an increase in SP. This raises some questions about the potential effect of solvent polarity of SCD with changes in its properties on the mechanism of pore formation with resins [47]. Coupled with the effect of dipolar interactions for Hansen’s three-component solubility parameter, an improved interpretation of the latter mechanism may be possible, and illustrates another knowledge gap to drive further research to address such questions.

## 6. Property Changes

### 6.1. Porosity

The variation of the surface area, pore volume and pore sizes for different types of PCR materials due to variations in porogenic solvents was discussed in this review with limited commentary on PSD. The tuning of PCR materials can yield three categories of polymers; micro-, meso- and macroporous resins. Figure 2 in ref. [16] shows an example from non-solvating pure solvent (2-ethylhexanol) and a solvating diluent (toluene), respectively [16]. The PSD characteristics of PCR materials are clearly seen by the shift from a bimodal to a unimodal distribution. The general mechanism of pore formation speculates that larger pores are formed for non-solvating diluents over solvating diluents, but not for bimodal size distributions. One may speculate that the SP does not remain constant but varies continuously during polymerization when mixtures of solvents undergo phase separation, resulting in bimodal distributions. In contrast, the addition of a non-solvating diluent (DMF) to 2-ethylhexanol causes a shift from a bimodal to a unimodal distribution (*cf*. Figure 3 in ref. [16]). The conclusion is speculative since there is strong support that solvent properties apart from SP (although dispersive, dipolar interactions and H-bonding are included in Hansen’s model), are generally unaccounted for in many studies. Therefore, solvent/monomer and solvent/polymer interactions need to be further studied in a systematic fashion to understand the pore formation mechanism. Unimodal and bimodal behaviour was similarly observed in other studies for different PCR materials [27,43]. With the aid of tuning experimental variables, as discussed herein, tunable bimodal distribution may be achieved.

### 6.2. Morphology

**Figure 9:**
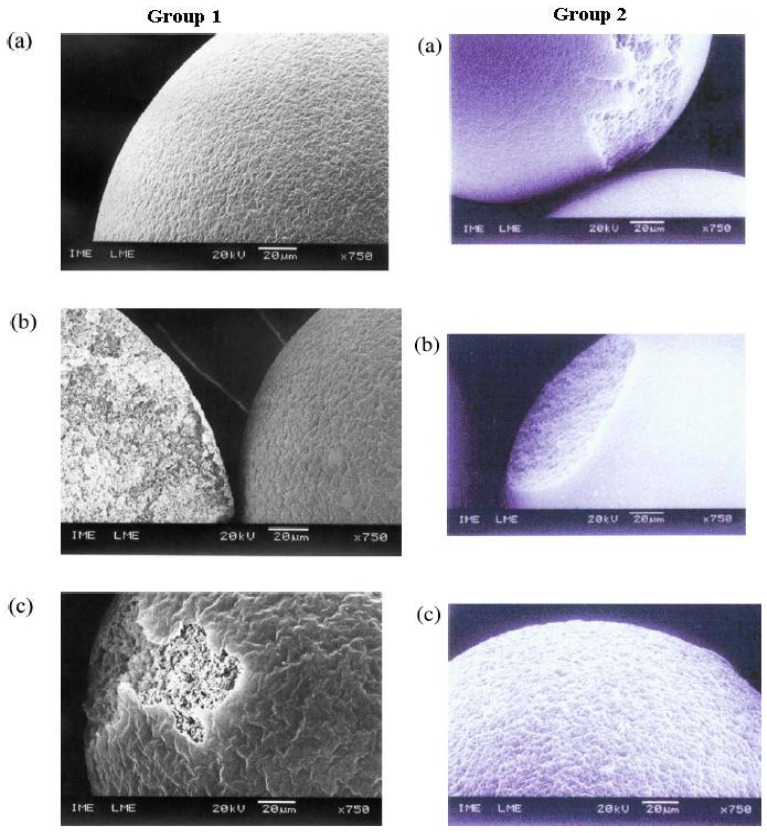
**Group 1**: SEM micrographs of the resins prepared with 150 % dilution using *n*-heptane/toluene as a diluent system at variable 2-vinyl pyridine/styrene/divinylbenzene ratios; (**a**) 30/4030;(**b**) 40/30/30 and (**c**) 50/20/30. **Group 2**: SEM micrographs of the resins synthesized with 100% dilution using *n*-heptane/ethyl acetate as a diluent system at 2-vinyl pyridine/styrene/divinylbenzene ratios; (**a**) 30/4030;(**b**) 40/30/30 and (**c**) 50/20/30. (Reprinted from [48], Copyright 2004, with permission from Elsevier).

The morphological properties PCR materials have been reported in various ways. In one case, Ortiz-Palacios and coworkers [2] argued that solvating diluents retain their individuality as microgels (microspheres), according to the general pore formation mechanism of porogenic solvents. This phenomenon renders the appearance of the morphology of the resin as smooth from the outside and rough on the inside due to micropore formation. The opposite is true for non-solvating diluents where the interior appears dense due to the lack of pore structure. On the contrary, de Santa Maria and coworkers [48] argue that a solvating diluent will increase porosity; hence, the surface of the resins will appear rough in appearance. There are differences in the appearance of PCR-based materials on the outer surfaces according to the nature of the solvent. The apparent contradiction may be due to the chemical type of PCR material. Ortiz-Palacios and coworkers [2] studied the styrene-*co*-divinylbenzene resin while de Santa Maria *et al*. [48] studied 2-vinylpyridine-*co*-styrene-co-divinylbenzene. In support of this, Lin and coworkers [49] used a solvating diluent which resulted in a smooth and regular surface. The appearance became rough upon addition of another copolymer. Figure 9 illustrates an example of the rough (Group 1) and smooth surfaces (Group 2) of the PCR materials at variable porogen composition. This observation was recently explained by Scheler [50] where he concluded that Hansen’s SP value changed during polymerization due to changes in the composition of the porogenic solvent mixture. The affinity between the resins and the evolving solvent composition, decreases or increases, during polymerization. This leads to differences in the morphology of the PCR materials.

## 7. Hansen Solubility Parameter

The three-component Hansen’s SP is a more useful criterion relative to the one- and two-component SP, as concluded by Rabelo and Coutinho [34]. In their research, they compared the one- and three-component SP values for the formation of styrene-*co*-divinylbenzene copolymer resins. Table 3 shows their findings and their classification of the solvents according to “good”, intermediary, and “bad” solvents using Equation (1) and (4) for the one- and three-component SP, respectively. Table 4 shows the textural properties of the resin for the solvents described in Table 3.

The resins with high surface area and low average pore diameter indicate a “good” solvent, *i.e.*, solvating diluent. The results in Table 3 infer that a one-component SP value does not adequately explain the properties of the resin, as shown in Table 4. In contrast, the three-component SP is a better predictor; however, some discrepancies are observed for diethyl phthalate, diisobutyl phthalate and dioctyl phthalate. These solvents yield mesopores with smaller size distributions than acetaphenone and toluene, as expected, according to the prediction of both one- and three-component SP values. The authors interpreted the results arising from differences in molar volume (*V_m_*) of the solvents, where *V_m_* is proportional to the enthalpy of mixing, as seen in Equation (7).


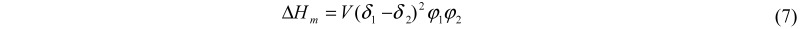


Δ*H_m_* is the enthalpy of mixing, *V* is the volume of the mixture, *φ*_1_ and *φ*_2 _are the volume fractions of the two porogens, and *δ*_1_ and *δ*_2 _are their respective SP values. As the magnitude of *V_m_* increases, the enthalpy of mixing increases which affects the free energy of mixing, as shown in Equation (8).


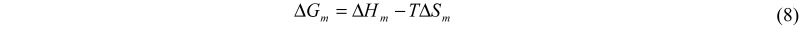


Δ*G_m_* is molar free energy of mixing, ΔS_m _is the molar entropy change of mixing, and T is the absolute temperature. Δ*S_m_* depends on the degree of polymerization, dispersion, aggregation and phase separation effects. An understanding of the enthalpy effects alone do not fully account for the interpretation of the trends observed because the value of Δ*S_m_* is also required for the process. As outlined in Section 5, other research has failed to adequately explain the observations using the SP approach until Scheler [50] suggested a potential explanation for the discrepancies. He observed that the solubility of the initiator such as AIBN dissolved in the porogenic solvent is equally important. Solvents that have comparatively low solubility for the initiator may influence migration of the initiator into the surface layers of the growing polymer nuclei, promoting further polymerization, and propagation. As a consequence, the interparticle voids are shrunken, and mesopores are converted into micropores.

**Table 3:** Prediction of porogenic solvents in terms of one- and three-component SP criteria; relative energy difference (RED) is calculated using Equation 3. (Data obtained from [34]).

One-component			Three-component		
Solvent	|Δδ| MPa^0.5^	Prediction	Solvent	RED	Prediction
Ethyl acetate	0.2	“Good” solvents *i.e.*, |Δδ| < 1.0	Acetophenone	0.28	“Good” solvents *i.e.*, |Δδ| < 1.0
Toluene	0.4		Diisobutyl phthalate	0.59	
Diisobutyl phthalate	0.4		Toluene	0.65	
Decaline	0.6		Diethyl phthalate	0.65	
Butyl acetate	1.2	Intermediary solvents *i.e.*, 1.0 |Δδ| < 3.0	Decaline	0.73	
Methyl-isobutyl ketone	1.4		Dioctyl phthalate	0.75	
Diethyl phthalate	1.8		Benzyl alcohol	0.87	Intermediary solvents, *i.e.*, 1.0 |Δδ| < 3.0
Isoamyl alcohol	1.9		Ethyl acetate	0.90	
Dioctyl phthalate	2.4		Butyl acetate	0.90	
Isoamyl acetate	2.6		Methyl-isobutyl ketone	0.94	
Acetophenone	3.1		Isoamyl alcohol	0.98	
Heptane	3.5	“Bad” solvents *i.e.*, |Δδ| > 3.0	Heptane	1.10	“Bad” solvents *i.e.*, |Δδ| > 3.0
Benzyl alcohol	6.1		Isoamyl alcohol	1.13	

**Table 4:** Porosity properties of styrene-*co*-divinylbenzene synthesized with different porogenic solvents. The parameters are obtained from nitrogen porosimetry (Data obtained from [34]).

Solvent	Fixed pore volume (cm^3^/g)	Surface area (m^2^/g)	Average pore diameter (nm)
Diethyl phthalate	0.47	149	12.6
Benzyl alcohol	0.65	110	23.6
Diisobutyl phthalate	0.57	83	27.5
Dioctyl phthalate	0.88	75	46.9
Heptane	0.99	68	58.2
Isoamyl acetate	0.34	30	45.3
Isoamyl alcohol	1.00	7	143
Toluene	0.01	0	–
Acetophenone	0.06	0	–
Methyl-isobutyl ketone	0.06	0	–
Decaline	0.12	0	–
Ethyl acetate	0.13	–	–
Butyl acetate	0.41	–	–

## 8. Knowledge Gaps

The tuning of PCR materials with porogenic solvents results in products with remarkable properties but there are some knowledge gaps identified herein that are recommended for further study. One of the gaps is the discrepancy of some results that are yet unaccounted for by the SP criteria. Although this has recently been addressed by Scheler [50] by accounting for the initiator solubility in porogenic solvent(s), it is anticipated to play a major role in the kinetics and thermodynamics of polymerization phenomena. The relationship between the initiator solubility and the resin properties require further research to support Scheler’s results. Rabelo and Coutinho [34] pointed out that molar volume prevails as a criterion of the SP since it is a measure of the solvating strength; however, the research is limited by the systematic correlations between V_m_ and SP. Secondly, the SP value is used for matching the resins and the solvent(s) at standard temperature conditions; however, polymerization often occurs at elevated temperatures ~60–80 ^o^C. Since the SP value is anticipated to change with temperature, a better matching of SP values should be calculated for *in-situ* polymerization temperature conditions [36,51]. Polarity is also temperature dependent; therefore, an improved knowledge of the solubility parameters for monomers, initiator, and polymer needs to be considered. Thirdly, the use of SP values to account for experimental results described in this review did not reliably address the accuracy of estimates for SP values of resin products. In general, estimates are typically obtained from the theoretical SP values in Hansen’s book [36]. Copolymers may behave differently depending on the nature of the co-monomers, relative composition, and the type of porogens employed [51]. There are different methods for estimating SP values of polymers including turbidimetry [52,53,54,55], intrinsic velocity [56], and the QSPR model [57]. The predicted results may vary considerably if more accurate estimates of SP values for the resins are used rather than the approximate values derived from tabulated values in handbooks. The accuracy of SP values has significant implication with respect to surface effect in PCR materials. It is generally underestimated in the literature how surface effects in PCR materials influence the pore formation mechanism, and should be considered in future research. Fourthly, the relationship to the variable morphology of the resins is not clear and whether the types of co-monomer, porogen, or both, are determinant factors. Further systematic studies are required to establish correlations. Finally, the relative influence of the thermodynamics and kinetics of pore formation in the formation of PCR materials is poorly understood at present, and further research is required to develop a more detailed understanding. One may assume that the formation of PCR materials is kinetically controlled by the slow separation of the constituent phases in favor individual microgel/microsphere domains. The other assumption is that it is governed by thermodynamic effects since the SP value is a key criterion for determining the final properties of the resin. 

## 9. Conclusions

Porous copolymer resin (PCR) materials are synthesized via suspension polymerization with variable textural properties by tuning the polymerization reaction with solvents which act as porogens. Porogenic solvents yield significant changes in the surface area, porosity, pore volume and morphology of the polymeric materials. The changes in physicochemical properties are attributed to the mutual solubility characteristics of the solvents, monomer units, and the polymeric resins. The closer the match of their mutual solubility parameters results in PCR materials with smaller pores and greater surface area. PCR materials with opposite characteristics are found for solvents with poor immiscibility and solubility.

Solvent tenability is possible by varying the relative solvent composition for mixtures, in accordance with Young’s rule. The solvent effect observed for PCR materials is thermodynamic (*i.e*., enthalpic) in nature and strongly correlates with the relative molar volume of the porogen solvent. The relative porogen size is not a comprehensive criterion for predicting the porogen properties of low molecular weight solvents; however, size effects are observed for oligomeric solvents such as PPG. Hansen’s three-component SP offers a better criterion for explaining the choice of the solvent system for yielding resin materials with different pore-structure properties. In some cases, there are discrepancies in the pore structure properties which may be attributed to the non-ideality of mixing (*i.e*., enthalpy and volume). Also, the importance of initiator solubility, steric effects due to H-bonding, and the relative immiscibility of monomer units are important considerations. Insolubility of the initiator is a recent finding that was unreported prior to 2007. Its importance relates to kinetic effects in the mechanism of formation of PCR materials. Hansen’s three-component SP provides a thermodynamic basis for understanding the mechanism of PCR-based materials.

The variation of resin morphology has been ignored for years, and was recently attributed to the temporal variation of the solvent mixture composition as the polymerization process proceeds. The general mechanism does not explain the origin of the bimodal PSD for low molecular weight solvents, such as 2-ethylhexanol, wherein; the effect disappears upon addition of a second solvent. The occurrence of surface effects for immiscible components is anticipated to contribute to considerable variation in the PSD. Therefore, the nature of solvent/polymer, solvent/monomer(s) and solvent/initiator interactions need to be further understood, and their relationship to the stabilization of the metastable structures, and final PCR products. Further research in this area will contribute to an improved understanding of the mechanism of the formation of PCR materials, and contribute to the development of tunable novel materials with versatile properties for a wide range of applications.
